# Referral and Final Diagnoses of Patients Assessed in an Academic Vertigo Center

**DOI:** 10.3389/fneur.2012.00169

**Published:** 2012-11-28

**Authors:** Rebekka Geser, Dominik Straumann

**Affiliations:** ^1^Department of Neurology, University Hospital ZurichZurich, Switzerland

**Keywords:** neuro-otology, vertigo, dizziness, diagnostic impact, benign paroxysmal positional vertigo, vestibular migraine, multisensory dizziness

## Abstract

**Objective:** To identify under-diagnosed neuro-otological disorders and to evaluate whether under-diagnosing depends on the age of the patient. **Materials and methods:** Retrospective analysis of medical charts from 951 consecutive patients (685 under and 266 above the age of 65 years) who entered diagnostic procedures at the Interdisciplinary Center for Vertigo and Balance Disorders, University Hospital Zurich, Switzerland. Final diagnoses were compared to referral diagnoses. **Results:** Relative to referral diagnoses, the proportion of patients finally diagnosed with benign paroxysmal positional vertigo (BPPV) almost doubled both in younger (<65 year from 12.7 to 25.1%) and older patients (from 20.7 to 37.6%). Striking relative increases were found for the diagnoses multisensory dizziness in older patients (from 20.7 to 37.6%) and vestibular migraine in younger patients (1.8 to 20.2%). In both age groups, the proportion of patients with undetermined diagnoses was reduced by about 60% (younger: 69.8 to 9.8%; older: 69.2 to 12.4%) by the diagnostic procedures in the vertigo center. These changes were all significant (*p* < 0.05) in McNemar tests with continuity correction (2 × 2 tables: focused diagnosis vs. other diagnoses, referral vs. final). **Conclusion:** Significant changes of diagnoses can be expected by a specialized neuro-otological work-up. In particular, BPPV, multisensory dizziness, and vestibular migraine are under-diagnosed by referring physicians. This finding calls for better education of primary care takers in the field of neuro-otology.

## Introduction

Dizziness ranks among the most common medical complaints in the general population. The self-reported prevalence among the working population is ∼20% (Yardley et al., 1998), and increases with age (Chu and Cheng, 2007; Gopinath et al., 2009). The symptoms are often reported to be severe enough to constitute a handicap for daily activities (Yardley et al., 1998; Mendel et al., 1999; Chawla and Olshaker, 2006; Chu and Cheng, 2007). Moreover, with a frequency of close to 2%, dizziness ranges among the most common reasons for consulting a primary care physician. Nearly 45% of outpatients with dizziness are seen and treated by general practitioners or family physicians (Sloane, 1989).

Dizziness as a non-specific symptom can be caused by a variety of disorders. These include peripheral vestibular disorders (e.g., benign paroxysmal positional vertigo, BPPV), central vestibular disorders (e.g., Wallenberg’s syndrome), cardio-vascular disorders (e.g., orthostatic arterial hypotension), ocular disorders (e.g., double vision due to ocular motor nerve palsy), somatosensory disorders (e.g., polyneuropathy), and others.

Bedside testing is the principal diagnostic procedure for evaluating patients suffering from vertigo. In selected cases, auxiliary vestibular tests (e.g., caloric testing), blood works (e.g., autoimmune markers), or imaging (e.g., MRI) may be required, while in others clinical evaluation alone is sufficient (Colledge et al., 1996; Kanashiro et al., 2005; Craighero et al., 2011; Jahn and Dieterich, 2011).

Due to the vast number of disorders that may cause dizziness, neuro-otological diagnoses are considered to be very demanding in a primary care setting (Colledge et al., 1996). The most frequent disorders causing dizziness, such as BPPV, vestibular neuritis, Ménière’s disease, and vestibular migraine, are usually accessible to treatment (Kanashiro et al., 2005; Lopez-Escamez et al., 2005; Sajjadi and Paparella, 2008; Strupp and Brandt, 2009; Mendel et al., 2010; Strupp et al., 2011). Similarly, cerebro-vascular disorders, which are the most common central causes of dizziness, are treatable by acute, e.g., thrombolytic, or prophylactic, e.g., anti-thrombotic, measures (Karatas, 2008). These effective treatment options for many causes of dizziness and the urgency for detecting potentially dangerous underlying disorders justify the need for efficient and reliable diagnoses of dizzy patients.

Still, many general practitioners seeing patients with dizziness are doubtful whether referral to a neuro-otological center would in fact lead to a significant change of the diagnosis and therefore a better treatment of patients. So far, epidemiologic studies were restricted to dizzy patients seen either by general practitioners (Sloane, 1989; Nazareth et al., 1999) or in specialized neuro-otological centers (Kanashiro et al., 2005). However, to our knowledge, neuro-otological diagnoses made by general practitioners were not compared to those made by specialists.

In this retrospective study we asked whether the diagnosis of dizzy patients referred to an academic neuro-otological center significantly changed in the course of a specialized work-up. In particular, we were interested in systematic differences between referral and final diagnoses of dizzy patients and whether these differences were age-dependent. More broadly, we aimed to contribute to the debate on whether specialized neuro-otological centers have a diagnostic impact and hence whether referrals to such centers are justified.

## Materials and Methods

### Patients

The Interdisciplinary Center for Vertigo and Balance Disorders at University Hospital Zurich is a collaboration of four departments (neurology, ENT, psychiatry, physical therapy). Patients are referred by general practitioners, neurologists, and ENT specialists from the greater Zurich area, but also from other areas within Switzerland. Less than 10% of patients are referred by physicians within the University Hospital. The center also evaluates patients on request by public and private insurances. In exceptional cases, the center accepts self-referral of patients.

Every patient is seen by a resident of neurology or ENT, who takes the medical history and performs a comprehensive battery of neuro-otological bedside tests. Each visit is concluded by the attendance of a senior physician specialized in neuro-otology, who reviews the medical history and repeats critical bedside tests. This study is restricted to the data of patients seen by neurology residents in the vertigo center from April 2004 to March 2008 (*N* = 951). Based on the clinical findings and, if ordered, on additional test results, the residents were instructed to select the final diagnosis from a list of 23 neuro-otological diagnoses (Table 1). If more than one diagnosis was applicable, the clinically most relevant one was chosen. The selection of the diagnosis was checked by the supervising senior physician as he was revising the report of the resident. In rare cases, an explicit selection from the list of diagnoses was missing, which was made up *post hoc* by the authors after carefully reading the final report.

**Table 1:** **List of neuro-otological diagnoses**.

Neuro-otological diagnoses	Criteria
Unclear dizziness	Normal bedside and laboratory tests, no psychogenic factors
benign paroxysmal positional vertigo	Positive provocation maneuvers with positional vertigo and semicircular-canal specific positional nystagmus
Multisensory dizziness	Significant deficits of at least two of the three major sensory inputs to the orientation system (vestibular, visual, proprioceptive)
Central vertigo	Clinical and/or radiologic (MRI) signs for central lesion causing dizziness
Unilateral peripheral vestibular deficit	One-sided pathological head impulse test
Ménière’s disease	American Academy of Otolaryngology-Head and Neck Foundation Inc. (1995)
Vestibular migraine	Diagnostic criteria for migrainous vertigo (Neuhauser and Lempert, 2005)
Bilateral peripheral vestibular deficit	Bilateral pathological head impulse test
Psycho-physiological dizziness	Causal psychogenic factors, normal bedside, and instrumental tests
Others	

*“Unilateral peripheral vestibular deficit” includes vestibular neuritis. “Others” combines the diagnoses: presyncopal dizziness, perilymph fistula, ocular vertigo, presyncopal dizziness, afferent ataxia, dizziness after head trauma, vestibular paroxysmia, acoustic neuroma, canal dehiscence syndrome, mal de débarquement, ototoxicity, dizziness after cervical spine distorsion, vertigo in cervical pain syndrome*.

In addition, the authors assigned a referral diagnosis to each patient included in this study. The referral diagnosis was chosen from the same list of neuro-otological diagnoses used for the final diagnoses and was based on the referral letter to the vertigo center. In self-referred patients, the “referral diagnosis” was extracted from the most recent report of the physician seeing the patient for dizziness.

### Analytical procedures

Statistical analysis concentrated on the most frequent nine diagnoses from the list. The remaining 14 diagnoses were merged and designated as “others” (Table 1).

Referral and final diagnoses were compared using the McNemar’s test with continuity correction (2 × 2 tables: focused diagnosis vs. other diagnoses, referral vs. final) to show whether changes of diagnoses were significant.

## Results

**Figure 1:**
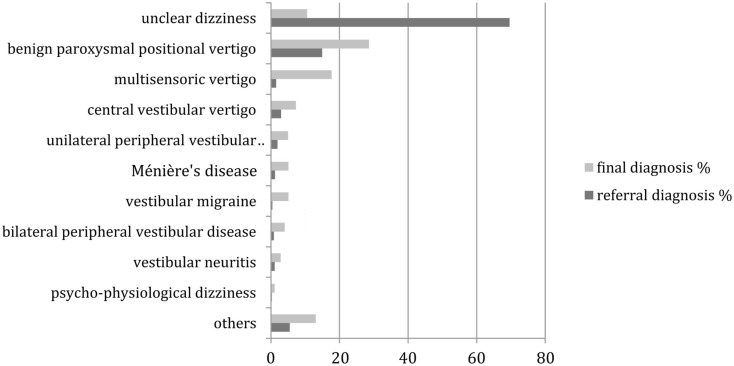
**Distribution of referral and final diagnoses in all patients**. “Others” include presyncopal dizziness, perilymph fistula, ocular vertigo, afferent ataxia, dizziness after head trauma, vestibular paroxysmia, acoustic neuroma, canal dehiscence syndrome, mal de débarquement, ototoxicity, dizziness after cervical spine distorsion, vertigo in cervical pain syndrome.

Of the 951 patients who entered diagnostic procedures at the vertigo center, 685 were under 65 years old and 266 were 65 years or older. Five hundred and six patients were female. Figure 1 compares the frequencies of referral and final diagnoses. The changes were all highly (*p* < 0.01) significant in the McNemar’s test. The largest difference was the reduction of “unclear dizziness” from 69.9 (662 patients) to 10.5% (100 patients). The frequency of all other diagnoses increased between referral and final. Most notably benign paroxysmal positional vertigo became the most frequent final diagnosis, as it increased from 14.9 (142 patients) to 28.6% (272 patients).

**Figure 2:**
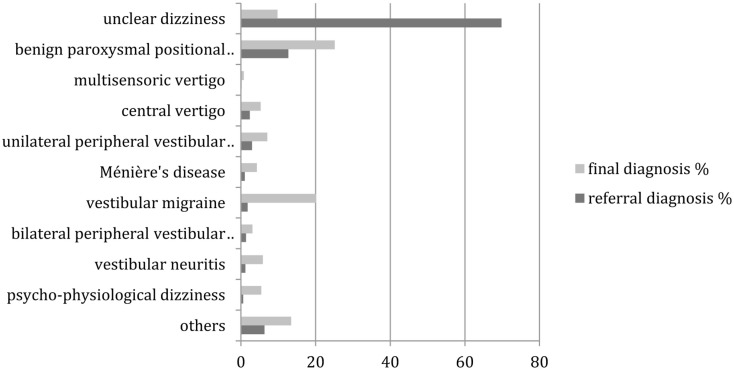
**Distribution of referral and final diagnoses in patients under the age of 65 years**. “Others” include presyncopal dizziness, perilymph fistula, ocular vertigo, afferent ataxia, and dizziness after head trauma, vestibular paroxysmia, acoustic neuroma, canal dehiscence syndrome, mal de débarquement, ototoxicity, dizziness after cervical spine distorsion, vertigo in cervical pain syndrome.

Since neuro-otological disorders are known to be age-dependent, we repeated the same analysis separately for patients younger than 65 years and for patients of age 65 years or above. Figure 2 shows the comparison between the referral and final diagnoses in the younger patients. Again, all changes were highly (*p* < 0.01) significant in the McNemar’s test. “Unclear dizziness” was reduced by 60% from 69.8 (478 patients) to 9.8% (67 patients), while benign paroxysmal positional vertigo increased from 12.7 (87 patients) to 25.1% (172 patients). A prominent relative increase was found for vestibular migraine from 1.8 (12 patients) to 25.1% (138 patients).

**Figure 3:**
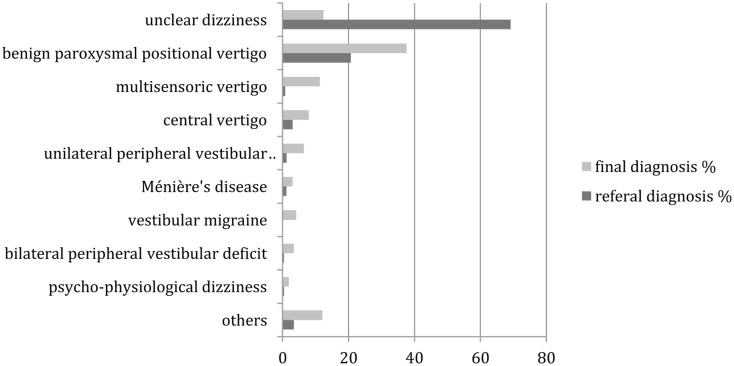
**Distribution of referral and final diagnoses in patients aged 65 years and above**. “Others” include presyncopal dizziness, perilymph fistula, ocular vertigo, afferent ataxia, dizziness after head trauma, vestibular paroxysmia, acoustic neuroma, canal dehiscence syndrome, mal de débarquement, ototoxicity, dizziness after cervical spine distorsion, vertigo in cervical pain syndrome.

Figure 3 provides the comparison between the referral and final diagnoses in patients aged 65 years or above. Again, most of the changes were highly (*p* < 0.01) significant in the McNemar’s test, except for Ménière’s disease, bilateral peripheral vestibular disease and psycho-physiological dizziness. As in the group of younger patients, “unclear dizziness” was reduced from 69.2 (184 patients) to 12.4% (33 patients), while “benign paroxysmal position vertigo” increased from 20.7 (55 patients) to 37.6% (100 patients). In contrast to the group of younger patients, multisensory dizziness increased from 0.8 (2 patients) to 11.3% (30 patients).

## Discussion

This retrospective analysis of patients referred to an academic vertigo center provides empirical evidence that a specialized neuro-otological work-up may lead to highly significant changes of diagnoses. Our findings show that practically all neuro-otological disorders are under-diagnosed at referral. Most strikingly, the proportion of patients diagnosed with “unclear dizziness” decreased from 70 to 10% as a result of the work-up. This was mainly due to a near doubling of the number of the patients diagnosed with benign paroxysmal positional vertigo, an increase seen in both the younger (<65 years) and the older (≥65 years) age groups. Moreover, striking relative increases of final diagnoses relative to referral diagnoses were found for multisensory dizziness in older patients and for vestibular migraine in younger patients.

This change of diagnoses by a neuro-otological work-up is of therapeutic relevance. If only benign paroxysmal positional vertigo, multisensory dizziness, and vestibular migraine were better diagnosed by such a specialized assessment, the proportion of patients receiving the appropriate treatment would already increase by one-third of all referred patients. Most patients with benign paroxysmal positional vertigo can successfully be treated with the appropriate repositioning maneuver for the affected semicircular-canal (Alvarenga et al., 2011; Do et al., 2011). Diagnosing multisensory dizziness in older patients leads to several helpful therapeutic measures such as physical therapy of balance, optimizations of eyeglasses, and the use of a cane (Strupp and Brandt, 2008). Finally, recognizing vestibular migraine in younger patients makes anti-migrainous substances a promising therapeutic option (Lempert et al., 2009; Furman et al., 2011; Strupp et al., 2011). Of course, most other neuro-otological conditions are also well treatable if correctly diagnosed (Kanashiro et al., 2005; Lopez-Escamez et al., 2005; Sajjadi and Paparella, 2008; Strupp and Brandt, 2008; Mendel et al., 2010).

History and bedside tests are crucial for finding the right diagnosis in dizzy patients as well as for differentiating between peripheral vertigo and central vertigo potentially in need of urgent therapeutic intervention (Kanashiro et al., 2005; Strupp and Brandt, 2008; Kattah et al., 2009; Ombelli et al., 2009). Considering that nearly 45% of outpatients with dizziness are seen and treated by general practitioners or family physicians (Sloane, 1989) and that our study demonstrates a significant change in diagnoses of vertigo after referral to a specialized center, underlines the importance of increasing neuro-otological skills of primary care physicians. In particular, these skills include bedside tests such as the head impulse test (Halmagyi and Curthoys, 1988) and maneuvers for detecting and treating BPPV (Fife, 2009). However, when diagnoses cannot be established with bedside tests alone, a referral to a specialized center should always be considered.

Finally, limitations of this study need to be mentioned: The patients seen by the neurology residents in the Interdisciplinary Center for Vertigo and Balance Disorders at University Hospital Zurich are by no means representative of the population of patients with dizziness in Switzerland. The consecutive patients retrospectively enrolled in our study were generally referred to the center because the referring physician was not able to provide a satisfactory treatment of their dizziness.

We also acknowledge the fact that referring physicians diagnose patients using heterogeneous criteria. These depend heavily on the quality of the training and continuing education. Thus a major factor leading to revised diagnoses are the application of consistent state-of-the-art diagnostic criteria in a vertigo center.

## Conflict of Interest Statement

The authors declare that the research was conducted in the absence of any commercial or financial relationships that could be construed as a potential conflict of interest.
